# Nutrition status and functional prognosis among elderly patients with distal radius fracture: a retrospective cohort study

**DOI:** 10.1186/s13018-020-01657-y

**Published:** 2020-04-07

**Authors:** Takako Nagai, Koji Tanimoto, Yoshiaki Tomizuka, Hiroshi Uei, Masahiro Nagaoka

**Affiliations:** 1 Department of Rehabilitation Medicine, Nihon University Hospital, 1-6 Kandasurugadai, Chiyoda-ku, Tokyo, 1018309 Japan; 2 Department of Orthopedic Surgery, Osumi Hospital, Tokyo, Japan; 3 Department of Orthopedic Surgery, Nihon University Hospital, Tokyo, Japan

**Keywords:** Distal radius fracture, Falls, Nutritional status, Activity of daily living, Geriatric

## Abstract

**Background:**

Distal radius fractures (DRF) are common in the elderly and are typically caused falls. Malnutrition has also been identified as a poor prognostic factor in elderly patients with fractures. However, the relationship between nutritional status and subsequent falls and functional prognosis for DRF in the elderly is not clear. The aim of the present study was to investigate the association between nutritional status and functional prognosis in elderly patients with DRF.

**Methods:**

Study participants included 229 outpatients who required surgical treatment for DRF. The patients’ clinical information, including age, sex, body mass index, bone mineral density, geriatric nnutritional risk index (GNRI), total number of drugs being treated with on admission, use of drugs for osteoporosis, comorbidity severity, the Barthel Index (BI), presence of subsequent falls, fracture type, postoperative follow-up period, and Mayo wrist score was reviewed. Subjects were further divided into two groups according to their GNRI: the malnutrition group and the normal group. Propensity score matching was used to confirm factors affecting the BI and subsequent falls.

**Results:**

Thirty-one patients (13.5%) presented with malnutrition before surgery for DRF. According to multiple liner regression analysis, the GNRI positively affected the efficiency of the BI (*β* = 0.392, 95% confidence interval [CI], 0.001 to 0.351, *p* = 0.039). Furthermore, on logistic regression analysis, subsequent falls were associated with serum albumin levels (odds ratio = 0.033, 95% CI, 0.002 to 0.477, *p* = 0.012).

**Conclusion:**

Malnutrition impaired improvement of activities of daily living (ADL) and increased the incidence of subsequent falls. Improvement of nutritional status before DRF surgery may further improve ADL and prevent falls.

## Background

Distal radius fracture (DRF) is the most common fragility fracture and is typically caused by a fall on an outstretched hand [1, 2]. There are two major age peaks for DRF: high-energy injuries deriving from sports injuries in young people and falls in older individuals [3]. As the life span of the general population increases, the incidence of DRF in the elderly is increasing [4]. In addition, elderly DRF patients are at high-risk of subsequent falls [5, 6]. It has been reported that decreased activity due to pain after DRF and fear of falling may cause a decline in muscle strength and balance, which may be a risk factor for future falls [7, 8]. A large clinical cohort of individuals followed for 10 years showed that those with a DRF had 11% more fractures than those without a prior fracture [9]. The association between DRF and subsequent fractures is independent of other osteoporosis risk factors. Non-modifiable factors such as age, sex, and prior fall history are well-established predictors of subsequent falls [10, 11].

More recently, bone density has been reported to be associated with risk factors for DRF and factors associated with falls after DRF [6, 12]. Low bone mineral density has also been reported in malnutrition [13, 14], while Adly et al. have reported that malnutrition increased the risk of falls [15]. However, there have been no reports describing the relationship between nutritional status and DRF or factors associated with falls after DRF.

This study hypothesized that there is an association between nutritional status and fall risk and impact on activities of daily living (ADL) after DRF. Further, the study examined the relationship between nutritional status after DRF and functional prognosis.

## Materials and methods

### Study design and participants

The retrospective study enrolled 229 patients aged 65 years or older presenting DRF admitted to an acute care hospital between October 2014 and December 2018 who underwent surgery and follow-up for at least 1 year after surgery. Patients were retrospectively identified via a search of the surgical database at our two affiliated hospitals. Demographic and postoperative clinical course information was extracted from each patient’s electronic medical record. Patients with neurological/cognitive impairment, multiple fractures, death, and missing data including bone density that could not be accurately measured after spine surgery were excluded. Ethical approval was obtained from each hospital’s ethics board. Patient informed consent was not required due to the retrospective design of the study.

### Surgical treatment and rehabilitation

All patients were treated with internal fixation using a volar locking plate (ACU-LOC plate, ACUMED, LLC, USA, *n* = 88 cases; Anatomic Volar Plate System, Depuy Synthes, Johnson-Johnson, Co., USA, *n* = 75 cases; Stellar2, HOYA Technosurgical Co., Japan, *n* = 42 cases; and APUTUS 2.5, Medical engineering system Co., Japan, *n* = 24 cases). A standard volar approach was used to expose the fracture side. The fracture was approached from the radial side of the flexor carpi radialis, and the quadrate pronator muscle was incised to reduce the fracture. If the fracture was unstable, it was reduced with Kirschner wire. Following the surgery, all patients were casted for 3–7 days depending on the stability of the fracture site. In postoperative rehabilitation, finger excursion training was started from the day after the operation. Active and passive training of the wrist joint with one-to-one guidance were started after cast removal.

### Measurements

Information collected for all patients included age, sex, body mass index (BMI), total number of drugs administered on admission, number and type of potentially inappropriate medications (PIMs) on admission, bone mineral density (as a percentage of the mean values for young adults), fracture type, comorbidity severity (Charlson comorbidity index, CCI), nutritional status (Geriatric Nutritional Risk Index, GNRI), wrist function criteria (Mayo wrist score), Barthel Index (BI), presence of subsequent falls, and follow-up periods after surgery.

Osteoporosis was defined as a T-score less than and equal to − 2.5 SD in the lumber vertebrae (L2–4). The AO classification was used to describe the DRF type. This classification system is commonly used for the radiographic classification of DRF and includes three types: A, B, and C. A is an extra-articular fracture, B is an intra-articular fracture, and C is an intra-articular complete fracture.

Comorbidity was assessed using the CCI [16]. The CCI is an indicator of multi-disease comorbidities and includes diabetes with chronic complications, heart failure, kidney disease, liver disease, chronic lung disease, dementia, hemiplegia or paraplegia, malignancy, and AIDS/HIV. The CCI uses a weighted score for each comorbidity, with higher numbers indicating a greater number of comorbidities and greater risk of mortality.

GNRI was calculated using the formula proposed by Bouillanne et al. [17]:

$$ 14.89\times \mathrm{serum}\ \mathrm{albumin}\ \left(\mathrm{g}/\mathrm{dL}\right)+\left\{41.7\ \mathrm{x}\ \left(\mathrm{current}/\mathrm{ideal}\ \mathrm{body}\ \mathrm{weight}\right)\right\}. $$

Individuals with GNRI less than 92 were assigned to the malnutrition group, and those with GNRI more than 92 were assigned to the normal group with mild or no risk of malnutrition.

ADL were evaluated by the BI. The BI is an assessment of 10 items: eating, moving, dressing, toilet movement, bathing, walking, going up and down stairs, changing clothes, defecation, and urination. Each item is scored as 0: unable to complete, 1: needs help, or 2: independent, and the total score is multiplied by 20, for a maximum score of 100. ADL was assessed before surgery and at the final follow-up. BI efficacy was defined by the BI at the end of follow-up minus the preoperative BI. The Mayo wrist score was used for wrist function evaluation. The scale includes scores for pain, functional status, range of motion, and grip strength, with a total score of 0 to 100. The higher the score, the better the function.

Subsequent falls were defined as falls caused by carelessness and did not include falls caused by traffic accidents, brain injuries, or diseases such as epilepsy.

### Outcomes

The primary outcome was BI gain, which was defined as the difference in the total BI at 1 year after surgery from that on admission. The secondary outcome was subsequent falls during follow-up periods.

### Statistical analysis

The subjects were divided into two groups: the malnutrition group and the normal group. The unpaired *t* test, Mann-Whitney’s *U* test, and *χ*^2^ test were used for comparison between the two groups, depending on variables and normality. Spearman’s rank correlation was used for univariate analysis of BI at final evaluation. Logistic regression analysis was also performed to determine whether the dependent variable was the presence or absence of a subsequent fall. As the number in the malnutrition group was small, the number of variables included in the logistic model had to be reduced. Propensity scores were calculated by logistic regression analysis including age, sex, comorbidity index, number of drugs, and fracture type as explanatory variables. All analyses were performed using IBM SPSS Statics version 25 (IBM Corporation; Armonk, NY, USA). A *p* value less than 0.05 was considered statistically significant.

## Results

During the study period, a total of 229 patients were eligible for participation. Patient characteristics are shown in Table 1. Thirty-one patients (37.2%) were in the malnutrition group. The malnutrition group had a lower BMI, serum albumin, and BI score (*p* < 0.001), and a higher number of subsequent falls and a CCI (*p* < 0.001, *p* = 0.006, respectively) than the normal group. Postoperative complications of DRF are shown in Table 2. In the malnutrition group, the rates of extensor pollicis longus (EPL) rupture, screw loosening, compression, and neuropathy were significantly higher than in the normal group. The cause of EPL rupture did not include, for the majority, screw dorsal cortex penetration or dorsal cortex comminution. The Spearman’s rank correlation results are shown in Table 3. GNRI was correlated with BMI, serum albumin, subsequent fall, BI at admission, and BI efficacy. BI efficacy was correlated with GNRI, serum albumin, and CCI, but not age, BMD and Mayo wrist score. There was no significant correlation between BMD and each factor, but the correlation coefficient was negative for CCI and fall during the follow-up period. Patients with complications and falls during the follow-up periods were considered to have lower BMD.

**Table 1:** Patients characteristics

	All (*n* = 229)	GNRI ≥ 92 (*n* = 198)	GNRI < 92 (*n* = 31)	*p* value
Age (year)	72.0 ± 8.1	76.1 ± 8.4	73.5 ± 8.1	0.770^1)^
Sex, female	198 (86.5)	168 (84.8)	30 (96.8)	0.194^2)^
Fracture type				0.903^2)^
AO type A	13 (5.6)	11 (5.5)	2 (6.5)	
Type B	82 (35.8)	70 (35.4)	12 (38.7)	
Type C	134 (58.5)	117 (59.1)	17 (54.8)	
BMI (kg/m2)	21.9 ± 3.9	22.8 ± 3.7	18.6 ± 2.6	< 0.001^1)^
Serum albumin (g/dl)	4.08 ± 0.38	4.18 ± 0.28	3.47 ± 0.37	< 0.001^1)^
BMD (g/cm^2^)	0.881 ± 0.143	0.885 ± 0.144	0.860 ± 0.136	0.364^1)^
CCI	1 (0–2)	1 (0–2)	1 (0–2)	0.006^2)^
Total number of drugs administered on admission	4 (0–14)	4(0–8)	5 (0–14)	0.274^1)^
Use of drugs for osteoporosis, *n* (%)	36 (15.7)	32 (16.2)	4 (12.9)	0.794^2)^
Days between onset and operation	4.81 ± 1.6	4.42 ± 2.1	5.18 ± 2.1	0.655^1)^
BI score				
Admission	77.2 ± 7.0	78.4 ± 6.3	69.8 ± 7.01	< 0.001^1)^
One year after surgery	86.4 ± 8.9	88.2 ± 7.8	75.2 ± 7.4	< 0.001^1)^
BI efficacy	9.2 ± 5.6	9.8 ± 5.6	5.3 ± 2.9	< 0.001^1)^
Mayo wrist score				
1 year after surgery	84.2 ± 6.6	84.3 ± 6.6	83.7 ± 6.8	0.645^1)^
Fall during follow-up periods, *n* (%)	21 (9.2)	9 (4.5)	12 (38.7)	< 0.001^2)^

Values are presented as mean ± standard, deviation, or number (%) or median (interquartile range).

*GNRI* Geriatric Nutritional Risk Indexes, *BMI* body mass index, *BMD* bone mineral density, *CCI* Charlson comorbidity index, *BI* Barthel Index, 1) Student *t* test, 2) chi-squared test

**Table 2:** Postoperative complications of DRF

Complications	All (*n* = 229)	GNRI ≥ 92 (*n* = 198)	GNRI < 92 (*n* = 31)	*p* value
				0.024^1)^
EPL rupture	3 (1.3)	2 (1.0)	1 (3.2)	
Screw loosening	1 (0.4)	0 (0)	1 (3.2)	
Compression	9 (3.9)	6 (3.0)	3 (9.7)	
Neuropathy	3 (1.3)	3 (1.5)	0 (0)	

Values are presented as number (%), *GNRI* Geriatric Nutritional Risk Indexes, 1) chi-squared test

**Table 3:** Spearman rank correlation coefficients among the factors

	Age	BMI	GNRI	Serum albumin	Total number of drugs on admission	CCI	BI at admission	BI efficacy	Mayo score	Fall during follow-up periods	BMD
Age	1	0.000	− 0.107	− 0.191^**^	0.276	0.090	− 0.126	− 0.089	− 0.124	0.169^*^	0.031
BMI	0.000	1	0.835^**^	0.193^**^	0.036	0.033	0.163^*^	0.033	0.002	− 0.115	− 0.028
GNRI	− 0.107	0.835^**^	1	0.701^**^	− 0.083	− 0.083	0.378^**^	0.206^**^	0.087	− 0.353^**^	0.028
Serum albumin	− 0.191^**^	0.193^**^	0.701^**^	1	− 0.194^**^	− 0.191^**^	0.462^**^	0.324^**^	0.152^**^	− 0.482^**^	0.032
Total number of drugs on admission	0.276	0.036	− 0.083	− 0.194^**^	1	0.303^**^	− 0.177^**^	− 0.053	− 0.161^*^	0.058	− 0.067
CCI	0.090	0.033	− 0.083	− 0.191^**^	0.303^**^	1	− 0.208^**^	− 0.180^**^	− 0.070	0.145^*^	− 0.058
BI at admission	− 0.126	0.163^*^	0.378^**^	0.462^**^	− 0.177^**^	− 0.208^**^	1	− 0.006	0.039	− 0.285^**^	− 0.044
BI efficacy	− 0.089	0.033	0.206^**^	0.324^**^	− 0.053	− 0.180^**^	− 0.006	1	0.055	− 0.105	0.098
Mayo score	− 0.124	0.002	0.087	0.152^**^	− 0.161^*^	− 0.070	0.039	0.055	1	− 0.101	0.098
Fall during follow up periods	0.169^*^	− 0.115	− 0.353^**^	− 0.482^**^	0.058	0.145^*^	− 0.285^**^	− 0.105	− 0.101	1	− 0.046
BMD	0.031	− 0.028	0.028	0.032	− 0.067	− 0.058	− 0.044	0.098	0.098	− 0.046	1

*BMI* body mass index, *GNRI* Geriatric Nutritional Risk Indexes, *CCI* Charlson comorbidity index, *BI* Barthel Index, *BMD* bone mineral density

*p < 0.05, **p < 0.01

The results of the multiple linear regression analysis for BI gain after propensity score matching for GNRI are shown in Table 4. Propensity scores were calculated by logistic regression analysis including age, sex, CCI, number of drugs, and fracture type as explanatory variables. GNRI positively affected the BI gain (*β* = 0.392, 95% confidence interval [CI] 0.001 to 0.351, *p* = 0.039).

**Table 4:** Liner regression analysis for BI efficiency

Variables	*β*	95% confidence interval		*p* value
		Lower	Upper	
PS	− 0.175	− 22.050	2.982	0.133
GNRI	0.392	0.010	0.351	0.039
Serum albumin	0.103	− 2.921	5.107	0.588

PS (log-transformed propensity score) was calculated from log transformation of the propensity score for age, sex, Charlson comorbidity index, number of drugs, and fracture type.

*GNRI* Geriatric Nutritional Risk Indexes

The results of the logistic regression analysis are shown in Table 5. The incidence of a subsequent fall was correlated with serum albumin (odds ratio 0.033, 95% CI 0.002 to 0.477, *p* = 0.012). Propensity scores were calculated by logistic regression analysis including age, sex, CCI, number of drugs, and fracture type as explanatory variables.

**Table 5:** Logistic regression analysis for subsequent fall

Variables	Odds ratio	95% confidence interval		*p* value
		Lower	Upper	
PS	18.987	0.018	19,590.442	0.406
GNRI	1.033	0.926	1.153	0.559
Serum albumin	0.033	0.002	0.477	0.012

PS (log-transformed propensity score) was calculated from log transformation of the propensity score for age, sex, Charlson comorbidity index, number of drugs, and fracture type.

*GNRI* Geriatric Nutritional Risk Indexes

## Discussion

The results of this retrospective cohort study revealed two aspects concerning nutrition status in patients with DRF. First, this study suggested that malnutrition was a risk factor for reduced ADL in older patients after DRF. Our results indicate that low serum albumin levels are associated with a risk of falling after DRF (Tables 3 and 5). This study supports the hypothesis that better nutritional status is associated with improved ADL for older patients after DRF. To our knowledge, this is the first study to show the impact of nutrition status on ADL in patients with DRF.

First, we found that malnutrition may lower the performance of ADL of older patients after DRF. There are several reports on the relationship between nutritional status and ADL, and poor nutritional status is associated with lower ADL [18–20]. Osta et al. reported that malnutrition was associated with lower education levels and older age, length of hospital stay, complications, multidrug use, and decreased ADL [18]. The prevalence of malnutrition in the elderly was reported to be 13.5% by Osta et al. and 17.9% by Krishnamoorthy et al. [19, 20]. Similarly, our study found that 13.5% of elderly DRF patients had malnutrition. Assessment of nutritional status after DRF may improve ADL by improving nutritional status

Second, we show that low serum albumin may increase the risk of a subsequent fall after DRF. In a previous study on fall risk, Mazur et al. reported that albumin <32 g/L, in addition to age ≥ 76 years, BMI < 23.5, Mini-Mental State Examination < 20, BI < 65, hemoglobin < 7.69 mmol/L, serum protein < 70 g/L, and calcium level < 2.27 mmol/L were risk factors for falls in hospitalized elderly patients [21]. Galet et al. reported that the rate of readmission hospitalizations among fallers increased from 15.6 to 17.4% between 2010 and 2014, proposing that social support and a fall prevention program are required [22]. In this study, GNRI, serum album, BI at admission, and CCI were identified as factors related to subsequent falls. Furthermore, a logistic regression analysis using propensity score matching for the probability of subsequent falls showed that serum albumin had an influence on the probability of subsequent falls. Hypoalbuminemia may be associated with falls because it leads to insufficient muscle synthesis and decreases in skeletal muscle mass, resulting in decreased balance and gait ability. These results suggest the necessity of introducing nutritional assessment and a fall prevention program in elderly DRF patients.

There was also no correlation between BMD and subsequent falls after DRF in this study. Patel et al. described the relationship between osteoporosis and falls as fall-related risk factors are common in older women referred for open access bone densitometry [23]. In this study, we examined the rate of falls after DRF, and it is possible that the lack of correlation between BMD and falls in our study was due to bias as patients with high activity levels had higher frequency of DRF. In addition, 15.7% of patients used medications to treat osteoporosis before the DRF, which may also have influenced the results. In the future, it is necessary to examine the relationship between osteoporosis drugs and bone density and subsequent falls.

This study has a few limitations. First, the assessment of muscle strength, gait function, balance, and postoperative nutritional assessment in patients with DRF was inadequate. In the future, it is necessary to examine the risk of falling by evaluating walking function, balance, and nutrition status during the postoperative follow-up period. Secondly, the verification of the life situation and the history of falls were insufficient, which should be verified in terms of their relationship with ADL.

## Conclusion

This study showed that malnutrition is related to the ability to resume ADL in elderly patients with DRF. Furthermore, low serum albumin levels may increase the risk of subsequent falls after DRF. Nutrition assessment and fall prevention programs may be of great benefit to older patients with DRF.

## Data Availability

The datasets used/or analyzed during the current study are available from the corresponding author on reasonable request.

## Abbreviations

BI: Barthel Index
BMI: Body mass index
BMD: Bone mineral density
CCI: Charlson comorbidity index
DRF: Distal radius fracture
GNRI: Geriatric Nutritional Risk Indexes
PS: Log-transformed propensity score
